# Supplementary material for: Multiple physiological and behavioural parameters identification for dietary monitoring using wearable sensors: a study protocol
Source: BMC Nutr. 2025 Oct 6;11:183. doi: 10.1186/s40795-025-01168-1 (PMC12502534; doi:10.1186/s40795-025-01168-1)
Supplement: Supplementary file 1 — Supplementary material 1 [file 40795_2025_1168_MOESM1_ESM.docx]

# **Appendix**

**Participant ID:**

**Meal Type:**

**Recorded by:**

| **Tick box** | **Timeline** | **Item** | **Time** | **Note** |
| --- | --- | --- | --- | --- |
|  |  | Rest for 10 min before the start |  |  |
|  | 5 min before baseline | Wearable Sensors:   - Put the wearable sensor on participant’s right hand, which is the dominant hand used for eating - Insert SD card into the wearable sensor - Turn the wearable sensor on until the light turns yellow - Check that the wearable sensor starts collecting data |  |  |
|  | 5 min before baseline | Bed-side Monitor   - Turn on the bedside monitor - Place blood pressure cuff on the right arm - Place pulse oximeter on the left hand   Measurements:   - Blood pressure 1 - Vital Signs 1 |  |  |
|  | Baseline 0 min | Baseline measurements:   - Blood pressure 2 - Vital Signs 2   Blood Collection 1 |  |  |
|  | From 0 min | Food consumption following the specific instructions |  |  |
|  | Finish eating | Measurements:   - Blood pressure 3 - Vital Signs 3 |  |  |
|  | 15 min | Blood Collection 2 |  |  |

**Participant ID:**

**Meal Number:**

**Recorded by:**

| **Tick box** | **Timeline** | **Item** | **Time** | **Note** |
| --- | --- | --- | --- | --- |
|  | 20 min | Measurements:   - Blood pressure 4 |  |  |
|  | 25 min | Measurements:   - Blood pressure 5 |  |  |
|  | 30 min | Measurements:   - Blood pressure 6   Blood Collection 3 |  |  |
|  | 35 min | Measurements:   - Blood pressure 7 |  |  |
|  | 40 min | Measurements:   - Blood pressure 8 |  |  |
|  | 45 min | Measurements:   - Blood pressure 9   Blood Collection 4 |  |  |
|  | 50 min | Measurements:   - Blood pressure 10 |  |  |
|  | 55 min | Measurements:   - Blood pressure 11 |  |  |
|  | 60 min | Measurements:   - Blood pressure 12   Blood Collection 5 |  |  |
|  | End | - Remove the blood pressure cuff and pulse oximeter - Turn off the wearable sensor and check data storage |  |  |

**Participant ID:**

**Meal Number:**

**Recorded by:**

| **Number** | **Timeline/min** | **Systolic BP 1** | **Diastolic BP 1** | **Systolic BP 2** | **Diastolic BP 2** | **Heart Rate** | **SPO2** | **Blood Collection** | **Time** |
| --- | --- | --- | --- | --- | --- | --- | --- | --- | --- |
| 1 | **-5** |  |  |  |  |  |  |  |  |
| 2 | **0** |  |  |  |  |  |  |  |  |
| 3 | **Finish eating** |  |  |  |  |  |  |  |  |
| / | **15** |  |  |  |  |  |  |  |  |
| 4 | **20** |  |  |  |  |  |  |  |  |
| 5 | **25** |  |  |  |  |  |  |  |  |
| 6 | **30** |  |  |  |  |  |  |  |  |
| 7 | **35** |  |  |  |  |  |  |  |  |
| 8 | **40** |  |  |  |  |  |  |  |  |
| 9 | **45** |  |  |  |  |  |  |  |  |
| 10 | **50** |  |  |  |  |  |  |  |  |
| 11 | **55** |  |  |  |  |  |  |  |  |
| 12 | **60** |  |  |  |  |  |  |  |  |
